# Cell-Free Scaffolds as a Monotherapy for Focal Chondral Knee Defects

**DOI:** 10.3390/ma13020306

**Published:** 2020-01-09

**Authors:** Haowen Kwan, Emanuele Chisari, Wasim S. Khan

**Affiliations:** 1 School of Clinical Medicine, Addenbrooke’s Hospital, University of Cambridge, Cambridge CB2 02P, UK; hk418@cam.ac.uk; 2 Department of Adult Hip and Knee Joint Reconstruction, Rothman Orthopaedic Institute, Philadelphia, PA 19107, USA; chisari.emanuele@gmail.com; 3 Division of Trauma & Orthopaedic Surgery, Addenbrooke’s Hospital, University of Cambridge, Cambridge CB2 0QQ, UK

**Keywords:** scaffold, knee, cartilage, cell-free, hydrogel, hybrid

## Abstract

Chondral knee defects have a limited ability to be repaired. Current surgical interventions have been unable to regenerate articular cartilage with the mechanical properties of native hyaline cartilage. The use of a scaffold-based approach is a potential solution. Scaffolds are often implanted with cells to stimulate cartilage regeneration, but cell-based therapies are associated with additional regulatory restrictions, an additional surgical procedure for cell harvest, time for cell expansion, and the associated costs. To overcome these disadvantages, cell-free scaffolds can be used in isolation allowing native cells to attach over time. This review discusses the optimal properties of scaffolds used for chondral defects, and the evidence for the use of hydrogel scaffolds and hydrogel–synthetic polymer hybrid scaffolds. Preclinical and clinical studies have shown that cell-free scaffolds can support articular cartilage regeneration and have the potential to treat chondral defects. However, there are very few studies in this area and, despite the many biomaterials tested in cell-based scaffolds, most cell-free studies focused on a specific type I collagen scaffold. Future studies on cell-free scaffolds should adopt the modifications made to cell-based scaffolds and replicate them in the clinical setting. More studies are also needed to understand the underlying mechanism of cell-free scaffolds.

## 1. Introduction

Cartilage is an avascular, aneural and alymphatic tissue, and knee cartilage damage has a limited ability to repair without surgical intervention [1]. If left untreated, the focal damage can lead to further degenerative changes and eventually progress to osteoarthritis [2]. Osteoarthritis is a degenerative disease characterised by cartilage erosion and low-grade joint inflammation, and is the leading cause of permanent disability [3]. Cartilage can be classified as fibrocartilage and hyaline cartilage. In the knee, the surfaces of the bone ends are covered with articular cartilage, a subtype of hyaline cartilage [4]. A chondral defect refers to a focal area of damage to articular cartilage and is currently managed with cartilage restoration surgical techniques.

A common feature of the different available cartilage restoration techniques currently used in clinical practice is the stimulation of cartilage regeneration through the use of cells and matrix remodeling [5]. Cartilage regeneration depends on chondrocytes that synthesise and maintain the cartilaginous matrix composed of collagens and proteoglycans. Currently, the most commonly used surgical interventions to regenerate cartilage include bone marrow stimulation, mosaicplasty, allograft transplantation and autologous chondrocyte implantation (ACI), and these are briefly outlined below.

Bone marrow stimulation is the oldest and most common method to stimulate cartilage regeneration [6]. The procedure involves penetrating and creating multiple openings in the subchondral bone plate (usually by microfracture), which allows bone marrow mesenchymal stem cells (MSCs) to migrate to the chondral defect site, proliferate and differentiate into chondrocytes to regenerate cartilage. However, the cartilage formed has been shown to be less mechanically stable and durable than natural hyaline cartilage [7]. Mosaicplasty involves removing cartilage from low-weight-bearing areas to fill the chondral defect site [8]. The residual gaps are then filled in by fibrocartilage, thereby forming a mosaic pattern. Allograft transplantation is a variation of this procedure in which the replacement cartilage is obtained from an allogeneic source. Nonetheless, there is debate as to whether the fibrocartilage tissue can provide sufficient stability [9]. In addition, the replacement cartilage often fail to integrate with the native cartilage, which hinders the healing process [4]. Autologous chondrocyte implantation (ACI) is a cell-based therapy which involves harvesting cartilage from low-weight bearing area, isolating chondrocytes, and expanding them in vitro [10]. Chondrocytes are then injected under a periosteal patch that covers the chondral defect. Clinical trials have shown that ACI can improve symptoms [11], and it has recently been approved by the National Institute for Health and Care Excellence (NICE) for symptomatic defects over 2 cm^2^ [12]. Despite this, cell-based techniques are costly and time-consuming as they require a two-step procedure: one to harvest chondrocytes and another to implant them. Biopsies taken to harvest chondrocytes also have the potential to cause donor-site morbidity [13]. Furthermore, the cartilage regenerated usually has limited mechanical properties [14], and a systematic review showed that ACI is not superior to microfracture or mosaicplasty for small defects [15].

Despite the many different treatments currently available, none of the procedures have been able to regenerate cartilage that mimics the mechanical properties of native articular hyaline cartilage [16]. The use of a scaffold-based approach is emerging as an innovative solution to the problem [17]. Its 3D structure can provide mechanical stability, and once inserted at the site of the chondral defect it acts as a platform for chondrocyte proliferation and matrix production [18]. Chondrocytes are usually cultured and implanted in the scaffold before it is inserted into the site of chondral defect. An example of this is matrix-induced ACI (MACI) which combines ACI with the use of a porcine 3D scaffold. Although potentially any cell source can be used, current clinical studies have focused on chondrocytes. Although MACI has produced promising clinical results [19], it still involves cell-based therapy and therefore carries the same inherent disadvantages in terms of needing a two-stage procedure and cell expansion, as well as the associated costs.

A more recent approach involves the application of scaffolds without cells [20], and allowing native cells from the knee joint to migrate and attach onto the scaffold [21]. As no cells are loaded in the scaffold before application, there is no need for a tissue harvest procedure or cell expansion thereby eliminating the possibility of donor site morbidity and the associated costs. This allows a single-stage treatment to be delivered at a fraction of the cost. It also avoids the ethical considerations and regulatory restrictions that, quite rightly, surround the use of cells. These advantages make cell-free scaffolds a promising alternative, but it is unclear how their effectiveness compares to existing interventions. This review will focus on the potential of cell-free scaffolds as a monotherapy in the management of chondral knee defects.

## 2. Optimal Properties of Scaffolds for Chondral Defects

The optimal scaffold should be biodegradable, biocompatible, mechanically stable and be able to provide the necessary environment for cells to attach, differentiate and produce cartilaginous matrix that in time replaces the scaffold [22]. A biodegradable scaffold is desirable as it would be completely degraded following cartilage regeneration and avoid an additional surgical procedure for its removal [23]. Scaffolds must also be biocompatible with the host to not cause any harm. To be biocompatible, they should be noncytotoxic and not elicit any undesirable systemic effects from the host. Some previously used synthetic scaffolds have been rejected and caused an immune response as their degradation products are recognised as foreign bodies [24]. Given the importance of these attributes, almost all biomaterials that are used to synthesise scaffolds are now both biodegradable and biocompatible. Studies that do not meet these criteria were not included in this review.

In addition, scaffolds need to be mechanically stable to support the load placed on the joint [25]. It has also been shown that the mechanical properties of the scaffold need to be similar to that of natural cartilage in order to cultivate cells [26]. However, the mechanical properties of cartilage are challenging to mimic as articular cartilage is composed of three different zones, each with its own organisation and properties [1,27].

Finally, for scaffolds to repair chondral defects, they must allow cells to attach onto the surface and regenerate articular cartilage [28]. The extent of cartilage regeneration is usually assessed by measuring the amount of collagen and proteoglycan produced. A significant challenge for cell attachment is cell distribution as cells tend to accumulate at the edges of the scaffold, leading to undesirable nonuniform cartilage formation. One of the critical factors affecting cell distribution is the size of pores. Smaller pores would limit the diffusion of nutrients and limits chondrogenesis to the peripheral boundaries only [29]. On the other hand, too large a pore would reduce the number of cells attached to the surface and may affect the mechanical properties of the scaffold [30]. It is essential to find the right balance to allow uniform cell distribution throughout the scaffold while maintaining mechanical stability. Many biomaterials have been studied as a candidate for scaffolds for chondral repairs [17], and below we discuss the two main groups of cell-free scaffolds that are based on hydrogel. Hydrogels are versatile scaffolds that are responsive to biological and mechanical stimuli, and can be used for additional modalities, e.g., localised drug delivery avoiding the side effects associated with systemic administration. The goal of using stimuli-responsive or multimodal hydrogels in clinical areas is still at the very early stage and advances in scaffold engineering are needed before this becomes a clinical reality including work on physical, chemical and biological properties.

## 3. Cell-Free Scaffolds

### 3.1. Hydrogel Scaffolds

Hydrogels, such as hyaluronan, fibrin and collagen, have hydrophilic surfaces which provide an excellent environment for cells to grow and preserve their native phenotype [23]. Of these hydrogels, studies on cell-free scaffolds so far have only focused on type I collagen (see Table 1). Collagen has been shown to facilitate chondrocyte proliferation while maintaining their phenotype [31] and is already commonly used in tissue engineering [32].

Schneider et al. [20] found that there is no significant difference in the quality of the regenerated cartilage between cell-free and cell-based scaffolds. The study used 18 adult Goettingen minipigs with full-thickness small chondral defects (6.3 mm), which were treated with either a cell-free collagen scaffold or a chondrocyte-seeded scaffold. Chondrocytes were observed in the cell-free scaffold after six weeks and a hyaline-like tissue was seen in both groups after one year. The quality of the regenerated cartilage was assessed histologically by O’Driscoll scoring and type II collagen staining, which showed no significant difference between the two groups. This result showed that cell-free scaffold has the potential to be a cheaper alternative to cell-based therapy without a reduction in quality.

Cell-free collagen scaffolds have also been investigated in clinical studies using type I collagen obtained from the tails of rats [33]. The study treated 15 patients with small chondral defects (<11 mm diameter) using this cell-free scaffold and evaluated the clinical and radiological results at a two-year follow up. The clinical outcomes assessed included pain reduction and patient activity. Both showed significant improvement after six months compared to preoperative values and continued to improve up to the two-year follow up. Radiological results measured by magnetic resonance imaging (MRI) showed that the scaffold had completely attached in 14 patients (93%) after six weeks. After two years, cartilage regeneration was observed as MRI showed that the defect had been completely filled with a homogenous structure with a smooth surface and nearly normal signal intensity.

While the previous study only measured short-term outcomes, Schüttler et al. [34] followed up on the same group of patients after four years to investigate the mid-term effects using the same variables. Both clinical and radiological outcomes continued to improve over time for every patient. Furthermore, the radiological appearance of the regenerated cartilage, as measured by MOCART score (Magnetic Observation of Cartilage Repair Tissue score), was comparable to those reported in studies using cell-based scaffolds [35], though this may be due to differences in methods. There are also some limitations with both studies as the quality of the regenerated cartilage was not assessed histologically and a larger sample size is needed.

In addition to small chondral defects, cell-free type I collagen scaffold has also been shown to be effective in patients with large chondral defects. Roessler et al. [36] treated 28 patients with a mean cartilage defect size of 3.71 cm^2^ and assessed their clinical and radiological outcomes at 6, 12 and 24 months. Pain reduction was noted after six weeks while patient activity almost reached preoperative levels after 12 months. Radiological results showed constant improvement from 12 months, and at 24 months, 24 patients (86%) showed homogenous structures completely filling the defect and integrating with bordering native cartilage. Moreover, the results of this study showed no significant difference to the clinical and radiological results of a cell-based type I collagen scaffold after two years [37].

Although the short-term result for large chondral defects was promising, a follow-up study showed a high risk of failure after five years in the same group of patients [38]. Of the 28 patients treated, five (18%) required revision surgery within five years. As before, all patients were evaluated clinically and radiologically. In addition, biopsies were taken during the revision surgery to histologically evaluate the failed regenerated cartilage. In patients with failed repair, MRI showed subchondral bone oedema which may be due to overload of the subchondral bone. Although histological assessment showed articular cartilage-like tissue, indicating successful cell colonisation and cartilage regeneration, there was also clustering of chondrocytes and inferior morphology which suggests inferior tissue quality. As for the remaining 23 patients, they continued to show good or even improved clinical results, but MRI showed a significant deterioration in radiological appearance between the two-year and five-year follow-up. This is particularly worrying as it suggests that the regenerated cartilage may not be able to handle the mechanical load placed on the joint and these patients may also require revision surgery in the future.

It seems that type I collagen scaffolds have the potential to be an effective treatment for small defects, but they may lack the mechanical properties to treat large chondral defects. This is perhaps unsurprising given that it is well documented that hydrogel scaffolds lack mechanical stability and are often unable to maintain their structures both in vitro and in vivo [39]. This may partly be due to the fast degradation rate of hydrogels, usually within days to weeks, which may not be sufficient for chondrocytes to regenerate stable cartilage [40]. A possible solution is to use stimuli-responsive hydrogel which degrades depending on various microenvironmental stimuli such as mechanical stretching and compression [41]. However, this type of biomaterial has not yet been applied to scaffolds treating chondral defects and future studies should investigate whether stimuli-responsive hydrogel offers superior mechanical properties.

### 3.2. Hydrogel–Synthetic Polymer Hybrid Scaffolds

Given the limitations of hydrogel scaffolds in addressing larger defects, alternative biomaterials for scaffolds have been explored. Synthetic polymers were considered as they are mechanically stronger and can be mass-produced with more precise properties and at a lower cost. Many different synthetic polymers were tested as potential scaffolds, including polyglycolic acid (PGA), polylactic acid (PLA), poly(lactic-co-glycolic) acid (PLGA) and poly(ethylene glycol)-terephthalate/poly(butylene terephthalate) (PEOT/PBT) [42]. Despite these efforts, cell-based scaffolds using only synthetic polymers have shown inadequate cell distribution due to a lack of hydrophilic surface [39], and the cells that attach onto the scaffolds often fail to form new cartilage [30]. Given the respective strengths and limitations of hydrogels and synthetic polymers, the most recent approach involves combining hydrogels and synthetic material. With the right balance, the hybrid scaffold would utilise the mechanical strength of synthetic polymers while hydrogels facilitate cell attachment and proliferation.

Despite the potential of hybrid scaffolds, very few studies have applied this to cell-free scaffolds. Even in cases where cell-free scaffolds are tested, they are only used as a control to compare against cell-based hybrid scaffolds. For example, Dai et al. [43] investigated the combination of type I collagen with polymer PLGA. The study tested both cell-based (seeded with bovine chondrocytes) and cell-free PLGA/collagen scaffolds which were transplanted subcutaneously into athymic nude mice. After eight weeks, mechanical stiffness for cell-based scaffolds reached 68% of that of natural cartilage. In addition, cell distribution was quite uniform and showed a cartilage-like phenotype on histology. On the other hand, cartilage formation in cell-free scaffolds was deficient and had almost no proteoglycan deposition. These scaffolds were also increasingly absorbed, with some almost disappearing after eight weeks. These initial results suggest that cell-free hybrid scaffolds may not be as effective as cell-based ones, but more studies on cell-free hybrid scaffolds are needed to confirm if this is the case. The study also highlights the need for further work on the hybrid scaffold biodegradation that may allow the scaffold to persist for long enough to allow for cell seeding and matrix formation.

## 4. Discussion

Preclinical and clinical studies have shown that cell-free scaffolds alone can support articular cartilage regeneration and have the potential to treat chondral defects. Nonetheless, there are few studies in this area, and it remains inconclusive whether it is as effective as cell-based scaffolds. The scarcity of evidence is compounded by small sample sizes in clinical studies and an almost exclusive focus on type I collagen scaffolds. Although cell-free scaffold is the most recent approach, the small number of studies is alarming since the first study focusing on cell-free scaffold was published in 2011. Since then, many studies have explored the possibility of modifying the cell-based hybrid scaffold to improve its mechanical properties and cell distribution [5], but this progress has not been replicated in cell-free scaffolds. A possible explanation is that cell-based scaffolds that utilise the more established ACI technique are considered more appropriate as they are focused on refining the current best practice in order to improve clinical outcomes rather than investigating the more radical cell-free scaffolds. Since cell-free and cell-based scaffolds have the same aim of chondrocyte proliferation on a 3D structure, it is likely that cell-free scaffolds would only match its cell-based equivalent at best and therefore make little difference in treatment outcome.

Although cell-free scaffold alone may not represent an upgrade on current treatment, it should still be considered as a potential alternative due to its cost-effectiveness. Cell-based scaffolds may be more advanced at the moment, but its high cost and time consumption means that it may not be economically feasible for all chondral defects to be treated in this manner. A study showed that although ACI has a slightly lower reoperation rate (12.1%) compared to microfracture (13.5%) after five years, microfracture is more cost-effective across all clinical outcomes due to its lower costs even when broader costs such as physiotherapies and work absences are included [13]. Similar studies comparing cell-free to cell-based scaffolds as well as other existing treatments are needed to determine cell-free scaffolds’ cost-effectiveness and whether it can be incorporated within the clinical treatment pathway for chondral defects.

Comparisons between cell-free and cell-based scaffolds have shown mixed results. The difficulty in making a conclusive comparison is due to the small number of studies and the differences in biomaterials used. The fact that studies on cell-free scaffolds have focused on type I collagen despite its well-known mechanical limitations again demonstrates how far cell-free scaffold is lagging in terms of research. Future studies on cell-free scaffolds should adopt the modifications made to cell-based scaffolds. For example, recent studies have explored the possibility of improving the structure of artificial hybrid scaffolds. To mimic the mechanical properties of natural cartilage as closely as possible, Ahn et al. [44] used mathematical modeling to determine an optimal structure for scaffolds. Based on the results of the analysis, the group used a custom-built machine to manufacture a 3D braid scaffold made of poly(lactic-co-glycolic acid) (PLGA) fibres and agarose gel matrix. This scaffold showed promising results both in terms of mechanical properties and cartilage regeneration but was only tested in scaffolds implanted with rabbit chondrocytes. Future research must keep up with the latest advances in scaffolds and replicate these in a cell-free setting to allow a fair comparison against the cell-based approach.

A better understanding of chondrocyte migration is also needed to determine whether cell-free scaffolds can be as effective as cell-based ones. While chondrocyte colonisation of the cell-free scaffold has been observed [20], the underlying mechanism of migration is yet unknown. Chondrocytes have been shown to migrate in vitro with the aid of chemoattractants, but this has not yet been proven in vivo [21]. Furthermore, it remains unclear where these chondrocytes migrate from. The adjacent native cartilage, the subchondral lamina and even sources outside the synovial fluid have all been suggested as potential sources of chondrocytes and it is possible that all of them contribute and there is no one primary source of chondrocyte during cartilage regeneration [36]. Given their ambiguous origins, it is also difficult to conclude whether chondrocyte migration occurs during cartilage regeneration or whether their progenitors, the MSCs, migrate onto the scaffold and then differentiate into chondrocytes. A study investigating PGA scaffold showed that some of the cells that attach onto scaffolds are multipotent which suggests that stem cells are involved in the regeneration process [45]. MSCs are usually activated in tissue injury and have been found in a wide range of tissues including cartilage, bone and bone marrow stroma [46]. Therefore, one possible mechanism is these MSC populations attach onto the scaffold, interact with the surrounding environment and then differentiate into chondrocytes [47]. Nonetheless, with so many unresolved questions regarding chondrocyte migration, more studies are needed to understand how cell-free scaffolds interact with chondrocytes and how this compares to chondrocyte implantation in cell-based scaffolds. We suggest the first line of enquiry to be the source of cells that colonise the cell-free matrices to lay down the cartilaginous matrix.

## 5. Conclusions

In summary, the limited current literature suggests that cell-free scaffolds can be effective as a monotherapy for chondral knee defects. However, more studies are needed to understand the underlying mechanism and determine whether it is a viable alternative to current treatments, especially cell-based techniques, both in terms of cost-effectiveness and treatment outcome. Future studies should adopt the modifications made to cell-based scaffolds and replicate them in a cell-free setting to enable a direct comparison.

## Figures and Tables

**Table 1:** Overview of studies testing cell-free hydrogel scaffolds.

Author(s)	Hydrogel Biomaterial	Type of Study and Sample Size	Type and Size of Defect	Follow-Up	Evaluations	Outcome
Schneider et al., 2011	Type I collagen	Preclinical on 18 Goettinger minipigs	6.3 mm full-thickness chondral defects	6, 12 and 52 weeks	Nondestructive biomechanical testing and histological evaluation including the O’Driscoll score	As assessed by O’Driscoll scoring and collagen II staining, repair tissue quality of the initially cell-free gel was equal to defects treated by cell-seeded collagen gel implantation after 1 year. After 1 year, a hyaline-like repair tissue in both groups has been created.
Efe et al., 2012	Type I collagen	Clinical study on 15 patients (6M, 9F) with a mean age of 26 (range: 19–40)	11 mm diameter	6 weeks, and 6, 12 and 24 months after surgery	Clinical: IKDC score, Tegner activity scale and VAS Radiological: MRI (6 weeks), MOCART score (at 6, 12 and 24 months)	The mean VAS after 6 weeks and the mean IKDC values after 6 months were significantly improved from the preoperative values. Significant improvement of the mean MOCART score was observed after 12 months. MRI at 24 months demonstrated complete filling in all cases with a mainly smooth surface, complete integration of the border zone, homogenous structure of the repaired tissue and nearly normal signal intensity.
Schüttler et al., 2014	Type I collagen	Clinical study on 15 patients (6M, 9F) with a mean age of 26.4 (range: 19–40)	11 mm diameter	6 weeks, and 6, 12, 24, 36 and 48 months after surgery	Clinical: IKDC score, Tegner activity scale and VAS Radiological: MOCART score	The mean VAS improved significantly when compared to the preoperative values. IKDC values increased significantly at final follow-up. After 36 months, a significant improvement was noted in the median Tegner score that lasted at least up to 48 months. The MOCART score improved consistently up to 4 years after implantation, with significant improvements already observed after 12 months.
Roessler et al., 2015	Type I collagen (CaReS-1S^®®^, Arthro Kinetics AG, Krems/Donau, Austria)	Clinical study on 28 patients (17M, 11F) with a mean age of 34.6 years	3.71 ± 1.93 cm^2^	6, 12 and 24 months after surgery	Clinical: IKDC score, Tegner activity scale, KOOS and VAS Radiological: MRI and MOCART score	Significant improvement in VAS scores after 6 weeks, and in IKDC and Tegner score from 12 months. All subject categories of the KOOS except for symptom (swelling) showed significant improvements. Significant improvements of the mean MOCART score from 12 months. MR images did not yield any signs of infection or synovitis. After 24 months a complete defect filling could be noted in 24 out of 28 cases with a mainly smooth surface, complete integration of the border zone and homogenous structure of the repaired tissue.
Schüttler et al., 2019	Type I collagen (CaReS-1S^®®^, Arthro Kinetics AG, Krems/Donau, Austria)	Clinical study on 28 patients (17M, 11F) with a mean age of 34.6 years	3.71 ± 1.93 cm^2^	1, 2 and 5 years after surgery	Clinical: IKDC score, Tegner activity scale, KOOS and VAS Radiological: MRI and MOCART score Histological (in cases of revision surgery): ICRS II score	Increased wear of the repair tissue and clinical failure in 18% of cases at 5-year follow-up requiring revision surgery. Histologic evaluation of the repair tissue showed a cartilage-like appearance with no signs of inflammation or cell death but an overall medium tissue quality according to the ICRS II Score.While the remaining patients showed good-to-excellent clinical outcomes, the radiologic appearance of the repair tissue showed a reduction of the MOCART score between the 2- and 5-year follow-up.

Male = M, Female = F, International Knee Documentation Committee score = IKDC score, Visual Analogue Scale = VAS, Magnetic Resonance Imaging = MRI, Magnetic Observation of Cartilage Repair Tissue score = MOCART score, Knee Operative Outcome Score = KOOS, International Cartilage Repair Society = ICRS.
